# Association Between Oral Function Limitation and Frailty-Related Functional Impairment in Older Koreans: An Analysis of KNHANES 2024

**DOI:** 10.3390/healthcare14162609

**Published:** 2026-08-19

**Authors:** Mi Ra Lee

**Affiliations:** Department of Dental Hygiene, Hanseo University, Seosan 31962, Republic of Korea; leemra1@hanmail.net; Tel.: +82-41-660-1576

**Keywords:** frailty, KNHANES, life functioning, older adults, oral frailty, oral function limitation

## Abstract

**Background/Objectives**: Oral function limitation (OFL) may be associated with functional decline in older adults, but evidence from nationally representative data remains limited. This study examined the association between OFL and frailty-related functional impairment among older Korean adults using data from the 2024 Korea National Health and Nutrition Examination Survey (KNHANES). **Methods**: This cross-sectional study included 1247 adults aged 65 years or older with complete data on chewing discomfort, speech discomfort, remaining natural teeth, Life Functioning-10 (LF-10), falls, and bedridden status. OFL was defined as the sum of chewing discomfort, speech discomfort, and having fewer than 20 remaining natural teeth, with scores ranging from 0 to 3. Outcomes were LF-10 score, falls during the past year, and bedridden status during the past month. Complex-sample linear and logistic regression analyses were performed with sequential adjustment for sociodemographic factors, health behaviors, chronic diseases, and oral health-related factors. **Results**: The weighted mean LF-10 score decreased stepwise as the OFL score increased, from 90.99 in the OFL = 0 group to 77.64 in the OFL = 3 group. In the fully adjusted model, higher OFL scores were significantly associated with lower LF-10 scores. Severe OFL was associated with a higher likelihood of bedridden status; participants with OFL = 3 had approximately five times the odds of being bedridden compared with those with OFL = 0. No consistent association was observed between OFL and falls. **Conclusions**: Higher OFL scores were associated with lower life functioning and a higher likelihood of bedridden status among older Korean adults. Simple OFL indicators may serve as oral health markers associated with current frailty-related functional impairment in community settings.

## 1. Introduction

As population aging progresses rapidly worldwide, functional decline and frailty in older adults have emerged as major public health concerns that extend beyond individual health and increase the burden on healthcare systems [1]. Frailty is a complex condition characterized by reduced physiological reserve accompanied by declines in mobility, self-care, and activities of daily living, rather than by a single disease [2]. Various instruments are used to assess frailty, including the physical frailty phenotype, the Frailty Index, and the Clinical Frailty Scale (CFS), which evaluate frailty on the basis of physical manifestations, accumulated health deficits, and overall clinical status, respectively [3,4,5]. These frailty assessment tools have been reported to be associated with health outcomes such as falls, disability, institutionalization, and mortality in older adults [3,4,5]. The 2024 Korea National Health and Nutrition Examination Survey (KNHANES) included the Life Functioning-10 (LF-10) to assess life functioning and functional ability in older adults [6]. The LF-10 comprises items related to activities of daily living and physical functioning, including rising from a chair, squatting, walking 400 m, climbing stairs, lifting objects, bathing or showering, using public transportation, and participating in social activities [6,7]. Although the LF-10 does not directly diagnose frailty or replace established frailty assessment tools, it can be used as an indicator of life functioning and functional health status that may be related to frailty [6,7]. Falls are a representative health outcome associated with functional decline and vulnerability in older adults [3,8], whereas bedridden status may be understood as an indicator reflecting activity limitation and reduced functional status in older adults [9].

Oral health is an important component of health that supports eating, speaking, living without pain, and participation in social activities, rather than merely denoting the presence or absence of teeth [10]. Older adults commonly experience a range of oral problems, including tooth loss, denture use, periodontal disease, dry mouth, decline in oral muscle function, and orofacial pain, which may be associated with chewing, speech, eating, communication, and social participation [10,11,12]. A recent meta-analysis of the global prevalence of oral and facial pain reported a pooled prevalence of 19.19% for concurrent oral and facial pain [13], underscoring the importance of pain in the oral and facial regions as an oral health concern. This epidemiological evidence supports evaluating oral status in older adults in relation to overall health and functional status rather than as an isolated dental concern.

More recently, the concepts of oral frailty and oral hypofunction have been introduced, and evidence has accumulated that mild declines in oral function may be associated with physical function, nutritional status, social functioning, and adverse health outcomes in older adults [11,14,15,16]. Tanaka et al. [14] defined oral frailty using a composite index that included the number of natural teeth, chewing ability, articulatory oral motor skills, tongue pressure, and subjective difficulties in eating and swallowing and reported that oral frailty was associated with adverse health outcomes, including mortality. In addition, systematic reviews, conceptual reviews, and concept analyses examining the determinants and conceptualization of oral frailty have strengthened the rationale for considering oral function decline an important health-management domain in aging societies [11,15,17]. In the present study, the oral function limitation (OFL) score was a pragmatic cumulative indicator constructed from three variables available in KNHANES: chewing discomfort, speech discomfort, and having fewer than 20 remaining natural teeth. It was not intended to replace established diagnostic instruments for oral frailty or oral hypofunction; rather, it was constructed to jointly reflect subjective oral function and objective dental status available in a large national survey. Chewing and speech discomfort represent subjective oral functional limitations experienced in daily life, whereas the number of remaining natural teeth represents objective dental status that supports oral function. Tooth number has been identified as an important oral health indicator associated with disability-free life expectancy and frailty [18,19]. In addition, the World Health Organization (WHO) [20] has proposed retention of at least 20 natural teeth as a criterion for functional dentition among adults aged 65–74 years, and retention of at least 20 natural teeth has been used as a criterion reflecting functional masticatory ability [21].

The association between OFL and functional status may involve nutritional, physical, and social factors. Chewing discomfort and a reduced number of remaining teeth may be associated with restricted food choices and lower dietary variety, which may in turn be related to poorer nutritional intake [22]. Such nutritional changes may be associated with reduced muscle mass and muscle function, as well as sarcopenia and physical functional decline; oral hypofunction has also been reported to be associated with frailty and sarcopenia [12]. Speech discomfort may be associated with communication difficulties and reduced social participation, and previous evidence linking oral frailty with social and physical frailty suggests the plausibility of such a social pathway [23]. Therefore, it is necessary to examine how an OFL indicator that jointly considers chewing discomfort, speech discomfort, and reduced tooth count is associated with life functioning and other indicators of functional impairment in older adults.

Accordingly, this study aimed to examine the associations of OFL score with frailty-related functional impairment indicators, including LF-10, falls during the past year, and bedridden status during the past month, among older Korean adults using KNHANES 2024 data. Because this study used cross-sectional data, it was designed to evaluate associations rather than the causal effects or predictive performance of OFL. The focus was to determine whether simple oral function indicators available in KNHANES may have relevance as oral health markers associated with the current functional status of older adults.

## 2. Materials and Methods

### 2.1. Participants

This study used data from the 2024 KNHANES conducted by the Korea Disease Control and Prevention Agency. KNHANES uses the most recent Population and Housing Census data available at the time of sample design as the basic sampling frame. Survey areas and households are selected as the primary and secondary sampling units, respectively, using a stratified cluster sampling design. All household members aged 1 year or older in sampled households are then included as survey participants [24].

**Figure 1:**
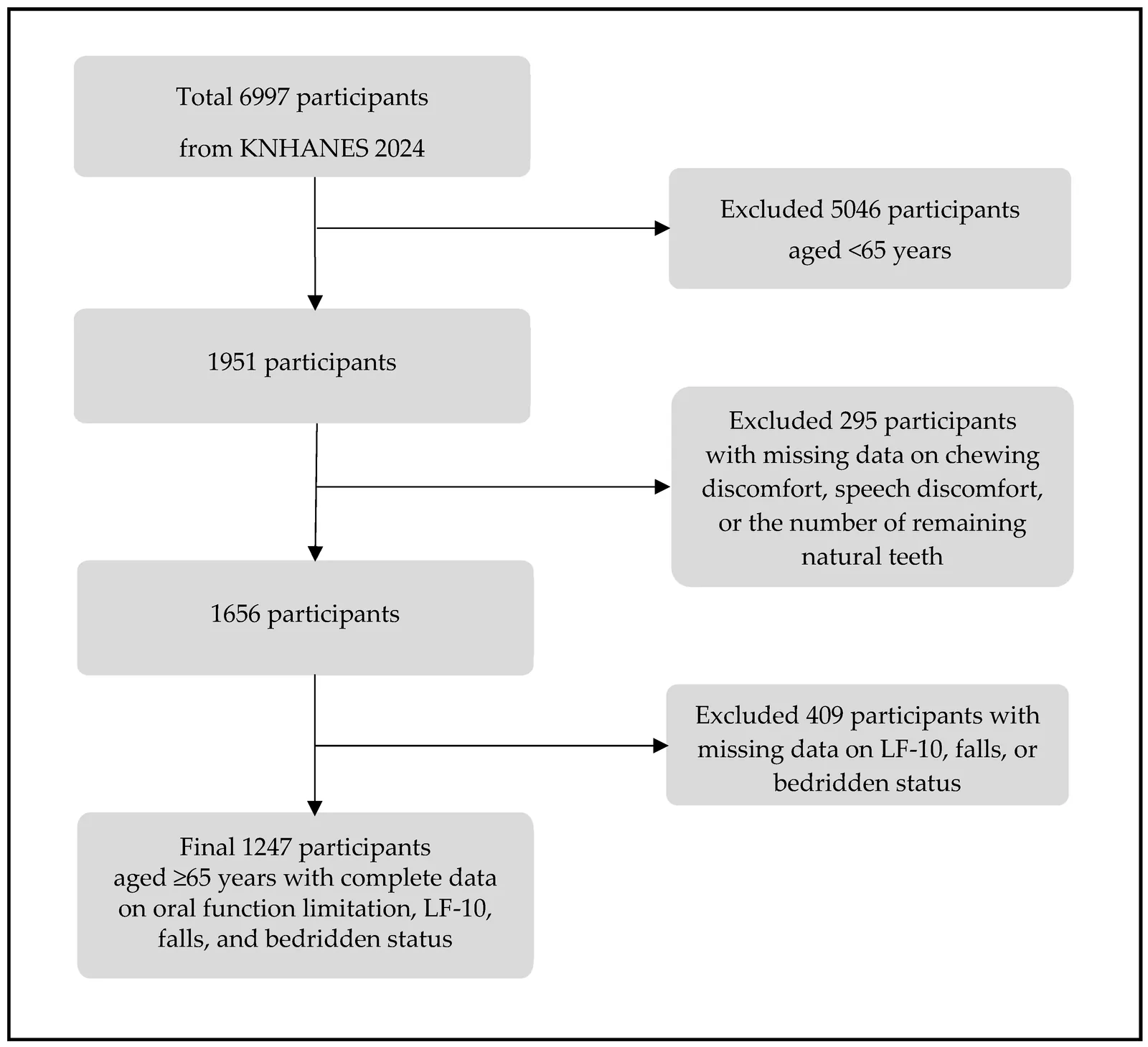
Flowchart of participant selection.

The inclusion criteria were age 65 years or older; responses to the chewing discomfort and speech discomfort items; oral examination data allowing determination of the number of remaining natural teeth; and complete responses for LF-10, falls during the past year, and bedridden status during the past month. The exclusion criteria were age younger than 65 years, missing oral function variables required to calculate the OFL score, or missing outcome variables. Among 6997 participants in KNHANES 2024, 1951 adults aged 65 years or older were initially identified. Of these, 1656 participants with sufficient data on chewing discomfort, speech discomfort, and remaining natural teeth to calculate the OFL score were retained. Finally, 1247 participants with complete data on LF-10, falls during the past year, and bedridden status during the past month were included in the analysis (Figure 1).

This study was a secondary analysis of publicly available, de-identified KNHANES data and was conducted after approval by the Institutional Review Board of Hanseo University (IRB No. HS26-0511-03).

### 2.2. Measurements

All questionnaire and measurement data used in this study were collected in Korean according to standardized KNHANES procedures. In this study, OFL was defined by combining chewing discomfort, speech discomfort, and the number of remaining natural teeth. The OFL score was not a validated scale intended to replace established diagnostic instruments for oral frailty or oral hypofunction; rather, it was a pragmatic cumulative indicator constructed from oral function-related variables available in KNHANES. Chewing discomfort and speech discomfort were assessed using the original KNHANES questions asking whether participants currently experienced discomfort when chewing food or speaking because of oral problems involving the teeth, dentures, gums, or other oral conditions [24]. For ease of analysis and clarity of interpretation, each item was recoded as a binary variable (no discomfort vs. discomfort). The number of remaining natural teeth was derived from oral examination data [24]. The threshold of fewer than 20 remaining natural teeth was established on the basis of the WHO criterion for functional dentition [20] and a previous epidemiological study that classified retention of at least 20 natural teeth as functional dentition [21]. The OFL score was calculated by assigning 0 or 1 point to each of three components—chewing discomfort, speech discomfort, and fewer than 20 remaining natural teeth—and summing the points to yield a score ranging from 0 to 3. Higher scores indicated a greater cumulative burden of oral function limitation. Because the three components reflect distinct domains—subjective chewing-related discomfort, subjective speech-related discomfort, and objective dental status—they were assigned equal weights and summed to quantify the cumulative extent of OFL. The OFL score is therefore a formative indicator reflecting the accumulation of different oral function domains, rather than a reflective scale in which multiple items repeatedly measure the same latent construct. Accordingly, an intraclass correlation coefficient (ICC) was not calculated because the components were not intended as repeated measures of a single latent construct.

The outcomes were LF-10 score, falls during the past year, and bedridden status during the past month as indicators of frailty-related functional impairment. The LF-10 is a life-functioning instrument developed and evaluated in community-dwelling Korean adults and reflects functional health status, including mobility, performance of daily activities, and social participation [6,7]. In this study, the LF-10 score calculated in KNHANES was used without modification as a continuous variable, with higher scores indicating better life functioning [6]. Falls were defined using the original KNHANES item on whether participants had experienced a fall during the past year and were categorized as yes or no [24]. Bedridden status was defined using the original KNHANES item on whether participants had spent almost the entire day in bed because of illness or injury during the past month and was categorized as yes or no [24]. Covariates were selected on the basis of previous studies and their availability in KNHANES 2024. Sociodemographic factors included age, sex, household income, education level, and marital status. Health behaviors and chronic diseases included smoking, drinking, body mass index (BMI), hypertension, and diabetes. Oral health-related factors included tooth brushing frequency, dental examination within the past year, and denture use.

### 2.3. Statistical Analyses

Because KNHANES uses a complex sampling design incorporating stratification, clustering, and sampling weights, all analyses in this study accounted for the complex survey design. The final analytic sample comprised 1247 participants with complete data for the main study variables; missing data were handled using complete-case analysis. Multiple imputation and separate sensitivity analyses were not performed. Because this study was a secondary analysis of an existing nationally representative survey, no a priori sample-size calculation or formal power analysis was performed.

Participant characteristics were presented as weighted percentages with standard errors, except for the total sample size, which was presented as an unweighted frequency. Differences in participant characteristics according to OFL score were examined using complex-sample chi-square tests. To describe frailty-related functional impairment according to OFL score, LF-10 scores were presented as weighted means with standard errors, whereas falls and bedridden status were presented as weighted percentages with standard errors. Differences in LF-10 score according to OFL score were examined using complex-sample general linear models, and differences in falls and bedridden status were examined using complex-sample chi-square tests. The association between OFL score and LF-10 score was analyzed using complex-sample linear regression, with regression coefficients (β) and 95% confidence intervals (CIs) reported. Associations of OFL score with falls and bedridden status were analyzed using complex-sample logistic regression, with odds ratios (ORs) and 95% CIs reported. In the primary analyses, OFL score was entered as a categorical variable with four levels (0, 1, 2, and 3), with OFL = 0 as the reference category. This approach allowed associations at each OFL level to be evaluated without assuming linearity. As a supplementary analysis, OFL score was entered as a continuous variable ranging from 0 to 3 to calculate *p* for trend and assess whether the associations showed a linear pattern across increasing OFL scores. Thus, the continuous OFL score was used only for trend assessment rather than for estimating the primary effect.

Regression models were constructed sequentially. Model I was unadjusted. Model II was adjusted for sociodemographic factors, including age, sex, household income, education level, and marital status. Model III was additionally adjusted for health behaviors and chronic diseases, including smoking, drinking, BMI, hypertension, and diabetes. Model IV, the fully adjusted model, additionally included oral health-related factors: tooth brushing frequency, dental examination within the past year, and denture use. Because denture use is related to both the number of remaining teeth and chewing function and may therefore also be associated with OFL score, Models III and IV were both presented to show changes in the estimates before and after adjustment for oral health-related factors including denture use.

To assess the adequacy of the regression models, multicollinearity between the main independent variable and covariates was examined. Variance inflation factors (VIFs) were calculated using a conventional linear regression model containing the same independent variables and covariates. VIF values for all variables ranged from 1.051 to 1.583, well below 10, indicating no serious multicollinearity. Model fit information provided by the SPSS Complex-Samples procedure was also reviewed to confirm the applicability of the complex-sample general linear and logistic regression models. Data were analyzed using IBM SPSS Statistics 20.0 (IBM Corp., Armonk, NY, USA), and statistical significance was set at a two-sided alpha level of 0.05.

## 3. Results

### 3.1. Participant Characteristics According to OFL Score

Table 1 shows participant characteristics according to OFL score. In the final analytic sample, 623 participants (50.0%) had OFL = 0, 435 (34.9%) had OFL = 1, 119 (9.5%) had OFL = 2, and 70 (5.6%) had OFL = 3. Higher OFL groups generally included a greater proportion of participants aged 80 years or older (*p* < 0.001), in the first to second household income quartiles (*p* = 0.002), with middle school education or less (*p* < 0.001), and who were divorced or widowed (*p* = 0.032). The proportions of non-drinkers (*p* = 0.002) and participants with diabetes (*p* = 0.035) were also higher in the higher OFL groups. In addition, tooth brushing fewer than three times per day (*p* = 0.020), no dental examination within the past year (*p* < 0.001), and denture use (*p* < 0.001) were more common in the higher OFL groups.

### 3.2. Frailty-Related Functional Impairment Indicators According to OFL Score

**Figure 2:**
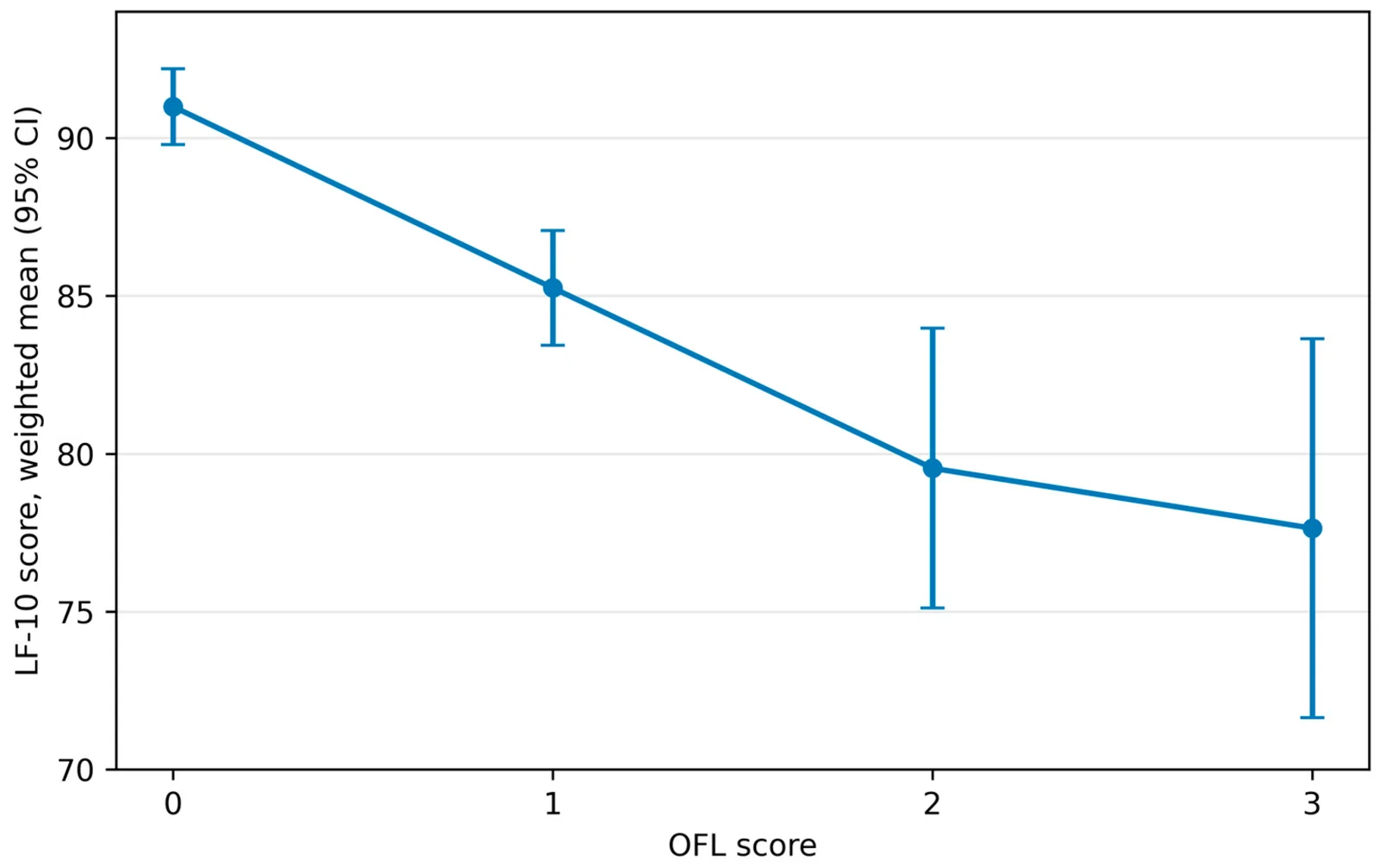
Weighted mean LF-10 score by OFL score with 95% confidence intervals. Note: Error bars indicate 95% confidence intervals. Unweighted sample sizes were 623, 435, 119, and 70 for OFL scores 0, 1, 2, and 3, respectively. OFL, oral function limitation; LF-10, Life Functioning-10.

Table 2 shows the LF-10 score, falls during the past year, and bedridden status during the past month according to OFL score. As the OFL score increased, the mean LF-10 score decreased stepwise from 90.99 in the OFL = 0 group to 85.25 in the OFL = 1 group, 79.54 in the OFL = 2 group, and 77.64 in the OFL = 3 group, with a statistically significant difference among groups (*p* < 0.001). The 95% CIs for the LF-10 score were 89.79–92.19 for OFL = 0, 83.43–87.07 for OFL = 1, 75.11–83.97 for OFL = 2, and 71.64–83.64 for OFL = 3 (Figure 2). Falls during the past year did not differ significantly according to OFL score (*p* = 0.727). In contrast, the prevalence of bedridden status during the past month increased from 4.1% in the OFL = 0 group to 5.4% in the OFL = 1 group, 7.5% in the OFL = 2 group, and 16.6% in the OFL = 3 group, and the difference among groups was significant (*p* = 0.001).

### 3.3. Association Between OFL Score and LF-10 Score

Table 3 shows the association between OFL score and LF-10 score. In complex-sample linear regression, higher OFL scores were associated with lower LF-10 scores from the unadjusted model (Model I) through sequentially adjusted models (Models II and III), and the *p* for trend was <0.001 in Models I–III. After full adjustment (Model IV), LF-10 scores remained significantly lower in OFL = 1, OFL = 2, and OFL = 3 compared to OFL = 0: OFL = 1 β = −2.17 (95% CI: −4.19, −0.15), OFL = 2 β = −4.48 (95% CI: −7.95, −1.01), and OFL = 3 β = −5.59 (95% CI: −11.08, −0.11). The *p* for trend in Model IV was 0.003.

### 3.4. Association Between OFL Score and Falls

Table 4 shows the association between OFL score and falls during the past year. No statistically significant association was observed in most models. In the fully adjusted model (Model IV), the OFL = 2 group had lower odds of falls than the OFL = 0 group (OR = 0.33, 95% CI: 0.12–0.92), whereas OFL = 1 and OFL = 3 were not significantly associated with falls. In addition, no significant linear trend was observed when the OFL score was entered as a continuous variable (*p* for trend = 0.209).

### 3.5. Association Between OFL Score and Bedridden Status

Table 5 shows the association between OFL score and bedridden status during the past month. In complex-sample logistic regression analyses, OFL = 3 was significantly associated with bedridden status in all models. In the fully adjusted model (Model IV), participants with OFL = 3 had approximately five times the odds of being bedridden compared with those with OFL = 0 (OR = 5.00, 95% CI: 1.58, 15.75), and the trend test using OFL score as a continuous variable was significant (*p* for trend = 0.023). In contrast, OFL = 1 and OFL = 2 were not significant in any model.

## 4. Discussion

This study examined the association between OFL and frailty-related functional impairment among older Korean adults using data from KNHANES 2024. The main findings were as follows. First, LF-10 scores decreased stepwise as the OFL scores increased, and this association remained after adjustment for sociodemographic factors, health behaviors and chronic diseases, and oral health-related factors. This finding suggests that an OFL score comprising chewing discomfort, speech discomfort, and reduced tooth count may be associated with overall functional health status in older adults, including mobility, performance of daily activities, and social participation, rather than reflecting only intraoral discomfort. Dibello et al. [11] suggested that oral frailty may be related to physical functional decline, and Tanaka et al. [14] reported an increased risk of physical frailty among individuals with oral frailty using a composite oral frailty index that included the number of natural teeth, chewing ability, articulatory oral motor skills, tongue pressure, and subjective difficulties in eating and swallowing. In addition, a longitudinal study of community-dwelling older adults in Korea by Kim et al. [25] reported changes in activities of daily living according to oral function level, supporting an association between oral function and life functioning. However, the OFL score used in the present study did not include the diverse objective oral function measurements included in the oral frailty index of Tanaka et al. [14] and should not be interpreted as equivalent to established diagnostic criteria for oral frailty or oral hypofunction. Accordingly, our findings should be interpreted not as a direct comparison with oral frailty diagnostic criteria, but as evidence that oral function-related indicators available in KNHANES—chewing discomfort, speech discomfort, and reduced tooth count—may be associated with current life functioning in older adults.

The observed association between OFL and reduced life functioning may be interpreted in the context of biological and social factors described in previous studies. Hoshino et al. [22] reported that the severity of oral frailty was associated with dietary variety among community-dwelling older adults; this finding may support the possibility that chewing discomfort and reduced tooth count are related to restricted food choices, lower dietary variety, poorer nutritional intake, and malnutrition. The reported association between oral hypofunction and sarcopenia [12], together with declines in masticatory muscle function and oral motor function described in the literature [26], may represent biological pathways relevant to the association between oral and physical function. Hironaka et al. [23] reported that oral frailty was associated with both social and physical frailty, providing indirect context for considering how limitations in speech function and communication difficulties may be related to social participation and life functioning. However, these nutritional, physical, and social factors were not directly assessed in this study; therefore, they should be regarded only as possible mechanisms for interpreting the association between OFL and functional impairment rather than as mechanisms established by the present data.

Second, participants with OFL = 3, representing severe oral function limitation, had a higher likelihood of bedridden status during the past month than those with OFL = 0. Bedridden status can be viewed as an indicator of functional impairment reflecting limitations in activities of daily living and physical function; however, studies directly examining the association between oral function decline and bedridden status remain limited. Kotronia et al. [27] reported that poor oral health was associated with disability and reduced physical function in older adults, and Watanabe et al. [16] reported that oral frailty was associated with increased mortality risk independently of physical and psychological frailty. Tamura et al. [28] further reported that posterior occlusal support or jaw stability provided by prostheses was associated with feeding and swallowing function in bedridden older adults. These studies suggest that oral health and oral function may be related to overall functional status and feeding or swallowing function in older adults and provide indirect context for interpreting the observed association between severe OFL and bedridden status. Nevertheless, because this study was cross-sectional, the findings cannot be interpreted as indicating that severe OFL causes bedridden status; they should be understood strictly as a statistical association between the two variables.

In contrast, falls during the past year did not show a consistent association with OFL level. In the fully adjusted model (Model IV), participants with OFL = 2 had lower odds of falls than those with OFL = 0, whereas no significant associations were observed for OFL = 1 or OFL = 3, and the p for trend was not significant. Therefore, the lower odds observed for OFL = 2 should not be interpreted as a protective effect of OFL. In the absence of an overall linear trend, this isolated finding may reflect chance variation arising from multiple comparisons, the limited sample size, or residual confounding. Although the meta-analysis by Satapathy et al. [29] reported an association between oral frailty and fall risk, falls in the present study were self-reported, and the OFL score did not include balance, muscle strength, occlusal force, tongue pressure, or other factors that may be directly related to falls. The findings for falls should therefore be interpreted cautiously as a secondary outcome.

A strength of this study is the use of nationally representative KNHANES 2024 data to examine the association between OFL and frailty-related functional impairment among older Korean adults. In addition, by combining subjective oral function indicators—chewing discomfort and speech discomfort—with an objective dental status indicator—fewer than 20 remaining natural teeth—the study evaluated the potential utility of a simple OFL indicator that can be derived from large-scale national survey data.

This study also has several limitations. First, because of its cross-sectional design, causal relationships and temporal ordering between OFL and functional impairment cannot be determined. Second, complete-case analysis was used; participants excluded because of missing data may have had poorer oral health or functional status than those included, and the absence of multiple imputation or sensitivity analyses means that selection bias cannot be ruled out. Third, chewing discomfort, speech discomfort, LF-10, falls, and bedridden status were based on self-reported data and may therefore be subject to recall or response bias. Fourth, because this was a secondary analysis of an existing national survey, no a priori sample-size calculation or formal power analysis was performed; moreover, limited sample sizes and event counts in some OFL subgroups and binary outcome analyses may have increased uncertainty in the estimates. Fifth, the OFL score did not include physiological indicators of oral hypofunction such as tongue pressure, occlusal force, dry mouth, swallowing function, or oral motor function and is not a screening tool with established diagnostic validity, sensitivity, specificity, reliability, or reproducibility. Sixth, interactions according to age, sex, denture use, or level of remaining tooth count were not examined. Seventh, important potential confounders—including depression, cognitive function, sarcopenia, medication use, chronic pain, nutritional status, and physical activity level—could not be fully accounted for, and residual confounding therefore remains possible.

Given these limitations, the present findings should not be interpreted as demonstrating that OFL predicts functional impairment or can be used as a clinical screening tool. Rather, they suggest the potential relevance of OFL as an oral health marker associated with current life functioning and bedridden status. Future studies should evaluate the validity, reliability, and reproducibility of the OFL indicator, compare it with established oral frailty or oral hypofunction assessment tools, and examine its prognostic value and temporal associations in longitudinal studies. Additional validation through prospective longitudinal and intervention studies is also needed before OFL-based assessment can be applied as a screening strategy in community settings.

## 5. Conclusions

This study examined the associations between OFL score and frailty-related functional impairment indicators among older Korean adults using data from KNHANES 2024. Higher OFL scores were associated with lower LF-10 scores, and severe oral function limitation (OFL = 3) was associated with a higher likelihood of bedridden status during the past month; however, no consistent association was observed with falls. These findings suggest that a KNHANES-based OFL score comprising chewing discomfort, speech discomfort, and fewer than 20 remaining natural teeth may have potential relevance as an oral health marker associated with current life functioning and bedridden status in older adults. However, because this study was cross-sectional and did not evaluate the predictive performance or diagnostic validity of OFL, the OFL score should not be interpreted as an established tool for screening older adults at high risk of functional impairment. Longitudinal and intervention studies are needed to evaluate the validity, reliability, reproducibility, concordance with established oral frailty assessment tools, and prognostic value of the OFL indicator.

## Figures and Tables

**Table 1:** Participant characteristics by OFL score.

Characteristics	Category	Total	OFL = 0	OFL = 1	OFL = 2	OFL = 3	*p*-Value
N	Unweighted n	1247	623	435	119	70	
Sex	Male	44.4 (1.3)	43.7 (1.8)	47.6 (2.5)	33.2 (4.4)	49.0 (6.9)	0.067
	Female	55.6 (1.3)	56.3 (1.8)	52.4 (2.5)	66.8 (4.4)	51.0 (6.9)	
Age (years)	65–74	62.1 (1.8)	69.7 (2.3)	58.5 (2.4)	40.5 (5.0)	50.3 (6.0)	<0.001
	75–79	20.1 (1.4)	18.4 (1.7)	20.9 (2.2)	28.7 (4.8)	17.2 (4.7)	
	≥80	17.8 (1.4)	11.9 (1.6)	20.6 (1.9)	30.8 (5.3)	32.5 (6.2)	
Household income	1st–2nd quartile	66.0 (2.0)	61.1 (2.6)	68.3 (3.0)	78.7 (4.0)	76.5 (6.1)	0.002
	3rd–4th quartile	34.0 (2.0)	38.9 (2.6)	31.7 (3.0)	21.3 (4.0)	23.5 (6.1)	
Education level	≤Middle school	63.3 (2.0)	56.5 (2.6)	66.7 (2.8)	80.9 (3.5)	75.4 (6.0)	<0.001
	≥High school	36.7 (2.0)	43.5 (2.6)	33.3 (2.8)	19.1 (3.5)	24.6 (6.0)	
Marital status	Married	69.0 (1.9)	72.9 (2.0)	67.4 (2.7)	56.9 (5.5)	62.4 (7.2)	0.032
	Single	1.1 (0.3)	1.2 (0.4)	0.9 (0.5)	0.6 (0.6)	2.0 (2.0)	
	Divorced or widowed	30.0 (1.8)	25.9 (1.9)	31.7 (2.7)	42.6 (5.6)	35.5 (7.1)	
Smoking	Yes	8.2 (0.9)	6.9 (1.2)	9.9 (1.7)	7.4 (3.4)	10.6 (3.3)	0.432
	No	91.8 (0.9)	93.1 (1.2)	90.1 (1.7)	92.6 (3.4)	89.4 (3.3)	
Drinking	Yes	66.6 (1.7)	68.9 (2.2)	69.5 (2.9)	47.9 (5.3)	57.1 (8.0)	0.002
	No	33.4 (1.7)	31.1 (2.2)	30.5 (2.9)	52.1 (5.3)	42.9 (8.0)	
BMI	Underweight	3.2 (0.6)	2.4 (0.7)	3.3 (0.9)	6.6 (2.8)	4.9 (3.1)	0.383
	Normal	32.1 (1.7)	32.0 (2.2)	31.1 (2.7)	33.4 (4.7)	38.3 (6.5)	
	Obese	64.7 (1.7)	65.6 (2.3)	65.6 (2.8)	60.0 (4.3)	56.8 (7.1)	
Hypertension	Normal	27.6 (1.5)	30.7 (2.1)	24.9 (2.4)	23.6 (4.9)	22.4 (5.5)	0.339
	Prehypertension	12.1 (1.0)	12.4 (1.4)	11.6 (1.7)	14.4 (4.2)	8.3 (3.7)	
	Hypertension	60.3 (1.4)	56.9 (2.0)	63.5 (2.6)	62.0 (5.5)	69.3 (6.1)	
Diabetes	Normal	27.7 (1.5)	27.3 (1.8)	27.2 (2.4)	29.3 (4.7)	31.4 (6.3)	0.035
	Impaired fasting	41.4 (1.5)	46.2 (2.2)	37.2 (2.7)	34.1 (5.0)	34.0 (6.5)	
	Diabetes	31.0 (1.5)	26.5 (1.9)	35.6 (2.9)	36.6 (5.0)	34.6 (6.1)	
Tooth brushing frequency	<3 times/day	44.5 (1.8)	41.7 (2.5)	44.1 (2.5)	50.7 (5.1)	62.8 (6.9)	0.020
	≥3 times/day	55.5 (1.8)	58.3 (2.5)	55.9 (2.5)	49.3 (5.1)	37.2 (6.9)	
Dental examination within 1 year	Yes	43.2 (1.7)	48.3 (2.2)	43.0 (2.9)	32.5 (4.2)	13.7 (4.9)	<0.001
	No	56.8 (1.7)	51.7 (2.2)	57.0 (2.9)	67.5 (4.2)	86.3 (4.9)	
Denture use	Yes	21.6 (1.5)	1.6 (0.5)	31.3 (2.7)	62.9 (5.5)	78.2 (5.7)	<0.001
	No	78.4 (1.5)	98.4 (0.5)	68.7 (2.7)	37.1 (5.5)	21.8 (5.7)	

Values are presented as weighted percentages (standard errors), except for unweighted sample size. OFL, oral function limitation; BMI, body mass index. *p*-values were obtained using complex-sample chi-square tests.

**Table 2:** Frailty-related functional impairment indicators by OFL score.

Outcome	OFL = 0	OFL = 1	OFL = 2	OFL = 3	*p*-Value
LF-10 score	90.99 (0.61)	85.25 (0.93)	79.54 (2.26)	77.64 (3.06)	<0.001
Falls	12.9 (1.3)	12.2 (1.8)	11.2 (2.8)	16.7 (5.2)	0.727
Bedridden	4.1 (0.9)	5.4 (1.1)	7.5 (2.6)	16.6 (5.3)	0.001

Values are presented as weighted means (standard errors) for LF-10 and weighted percentages (standard errors) for falls and bedridden status. LF-10 scores range from 0 to 100, with higher scores indicating better life functioning. OFL, oral function limitation; LF-10, Life Functioning-10. *p*-values were obtained using complex-sample general linear models for LF-10 and complex-sample chi-square tests for falls and bedridden status.

**Table 3:** Association between OFL score and LF-10 score.

Predictor	Model I β (95% CI)	Model II β (95% CI)	Model III β (95% CI)	Model IV β (95% CI)
OFL = 0	ref.	ref.	ref.	ref.
OFL = 1	−5.74 (−7.87, −3.61) ***	−3.90 (−5.87, −1.94) ***	−2.55 (−4.52, −0.59) *	−2.17 (−4.19, −0.15) *
OFL = 2	−11.45 (−15.84, −7.07) ***	−5.66 (−9.25, −2.07) **	−5.52 (−8.66, −2.38) **	−4.48 (−7.95, −1.01) *
OFL = 3	−13.35 (−19.25, −7.45) ***	−9.69 (−14.60, −4.78) ***	−7.12 (−12.46, −1.78) **	−5.59 (−11.08, −0.11) *
*p* for trend	<0.001	<0.001	<0.001	0.003

Values are presented as regression coefficients (β) and 95% confidence intervals (CIs). OFL, oral function limitation; LF-10, Life Functioning-10; CI, confidence interval; ref., reference. Model I was unadjusted; Model II was adjusted for age, sex, household income, education level, and marital status; Model III was additionally adjusted for smoking, drinking, BMI, hypertension, and diabetes; Model IV was additionally adjusted for tooth brushing frequency, dental examination within 1 year, and denture use. *p* for trend was calculated by entering OFL score as a continuous variable. * *p* < 0.05, ** *p* < 0.01, *** *p* < 0.001.

**Table 4:** Association between OFL score and falls.

Predictor	Model I OR (95% CI)	Model II OR (95% CI)	Model III OR (95% CI)	Model IV OR (95% CI)
OFL = 0	ref.	ref.	ref.	ref.
OFL = 1	0.93 (0.62, 1.40)	0.92 (0.60, 1.41)	0.83 (0.49, 1.38)	0.66 (0.37, 1.16)
OFL = 2	0.85 (0.47, 1.52)	0.68 (0.37, 1.24)	0.51 (0.21, 1.25)	0.33 (0.12, 0.92) *
OFL = 3	1.35 (0.64, 2.84)	1.34 (0.59, 3.01)	1.51 (0.52, 4.35)	0.85 (0.28, 2.59)
*p* for trend	0.824	0.880	0.823	0.209

Values are presented as odds ratios (ORs) and 95% confidence intervals (CIs). Falls were defined as at least one fall during the past year. OFL, oral function limitation; OR, odds ratio; CI, confidence interval; ref., reference. Model adjustments are the same as those described in Table 3. *p* for trend was calculated by entering OFL score as a continuous variable. * *p* < 0.05.

**Table 5:** Association between OFL score and bedridden status.

Predictor	Model I OR (95% CI)	Model II OR (95% CI)	Model III OR (95% CI)	Model IV OR (95% CI)
OFL = 0	ref.	ref.	ref.	ref.
OFL = 1	1.33 (0.76, 2.32)	1.19 (0.68, 2.08)	1.41 (0.69, 2.87)	1.43 (0.65, 3.14)
OFL = 2	1.87 (0.76, 4.60)	1.40 (0.58, 3.38)	0.78 (0.25, 2.38)	0.81 (0.26, 2.49)
OFL = 3	4.61 (1.95, 10.88) **	3.70 (1.48, 9.24) **	5.08 (1.72, 14.96) **	5.00 (1.58, 15.75) **
*p* for trend	0.002	0.018	0.029	0.023

Values are presented as odds ratios (ORs) and 95% confidence intervals (CIs). Bedridden status was defined as staying in bed for almost the entire day because of illness or injury during the past month. OFL, oral function limitation; OR, odds ratio; CI, confidence interval; ref., reference. Model adjustments are the same as those described in Table 3. *p* for trend was calculated by entering OFL score as a continuous variable. ** *p* < 0.01.

## Data Availability

The KNHANES data used in this study are publicly available from the Korea Disease Control and Prevention Agency website after user registration (URL: https://knhanes.kdca.go.kr/knhanes/main.do#; accessed on 20 January 2026).

## Abbreviations

The following abbreviations are used in this manuscript:

BMI	Body mass index
CFS	Clinical Frailty Scale
CI	Confidence interval
IRB	Institutional Review Board
KNHANES	Korea National Health and Nutrition Examination Survey
LF-10	Life Functioning-10
OFL	Oral function limitation
OR	Odds ratio
SE	Standard error
VIF	Variance inflation factor
WHO	World Health Organization
